# Evaluation of the new Egyptian undergraduate medical program from the perspectives of faculty and institutional leadership: a nationwide, mixed-method study

**DOI:** 10.1186/s12909-026-09865-6

**Published:** 2026-08-13

**Authors:** Somaya Hosny, Mona Ghaly, Asmaa Abdelnasser, Ahmed Makhlouf, Hala Salah, Abdelrahim Shoulah, Sanaa Radi, Zeinab Said, Hany Atwa

**Affiliations:** 1 Faculty of Medicine, Suez Canal University, Ismailia, 41522 Egypt; 2 Ibn Sina National College for Medical Studies, Jeddah, Kingdom of Saudi Arabia; 3 Faculty of Medicine, Assiut University, Assiut, Egypt; 4 Badr University in Cairo, Cairo, Egypt; 5 Faculty of Medicine, Cairo University, Cairo, Egypt; 6 Faculty of Medicine, Benha University, Banha, Egypt; 7 Faculty of Medicine, Alexandria University, Alexandria, Egypt; 8 Faculty of Medicine for Girls, Al-Azhar University, Cairo, Egypt; 9 College of Medicine and Health Sciences, Arabian Gulf University, Manama, Kingdom of Bahrain

**Keywords:** Undergraduate medical education, Program evaluation, Faculty perception, Curriculum reform, Competency-based education, Egypt

## Abstract

**Background:**

In response to global calls for medical education reform, Egypt restructured its national undergraduate medical education program from a 6 + 1 model to a competency-based “5 + 2” model. This reformed program emphasizes early clinical exposure, integration of basic and clinical sciences, student-centered learning, and alignment with national academic reference standards. With the graduation of the first cohort in 2023, this study aimed to evaluate the program’s effectiveness from the perspectives of faculty and institutional leadership.

**Methods:**

A concurrent mixed-methods design was employed. Quantitative data were collected through a validated, researcher-developed online survey distributed to faculty across 27 Egyptian medical colleges. A total of 10,728 responses were received. Exploratory factor analysis confirmed the construct validity of the survey, which comprised 42 items grouped into five domains: Program Goals, Content, Learning Resources, and Student Assessment; Program Impact on Students’ Professional and Research Skills; Faculty Involvement and Support; Students’ Academic Guidance and Support; and Physical and Learning Facilities. Qualitative data were obtained from nine focus group discussions (FGDs) involving 100 academic leaders. Thematic analysis of the FGDs offered complementary insights into program implementation.

**Results:**

Faculty responses indicated borderline to moderate overall satisfaction (mean score 2.99). Higher scores were reported for accessibility and relevance of learning resources and fairness of student assessment. Satisfaction was lower regarding opportunities for faculty to participate in curriculum design and institutional support for research. No significant gender-based differences were found. Junior faculty reported higher satisfaction than senior faculty (*p* < 0.001). Thematic analysis revealed the academic leaders’ appreciation for integrated teaching, early clinical exposure, and modern pedagogies, but also highlighted challenges such as inadequate infrastructure, faculty resistance to change, and variability in curriculum delivery. Leadership support, student feedback mechanisms, and faculty development were noted as essential enablers for successful implementation.

**Conclusions:**

This evaluation documented faculty and leadership perceptions of strengths in curriculum integration, learning resources, and assessment strategies, while also identifying critical implementation challenges. Ongoing faculty development, equitable institutional support, and sustained stakeholder engagement are essential to ensure the long-term success of the reformed program. Findings may offer insights for policymakers and educators working within similarly centrally coordinated, competency-based curriculum reform initiatives, particularly in contexts with comparable national governance structures.

**Supplementary Information:**

The online version contains supplementary material available at 10.1186/s12909-026-09865-6.

## Background

Over the last two decades, there has been a global push for transformative changes in medical education programs. In response, numerous medical schools worldwide have introduced updated undergraduate curricula to align with the contemporary needs of medical practice [1]. The necessity for reforms in medical education is increasingly recognized due to rapid advancements in healthcare technologies, shifting patient demographics, and the ongoing challenges posed by global health crises [2, 3]. Traditional medical curricula often struggle to address the growing demand for adaptable, interdisciplinary, and evidence-based approaches in medical practice [4].

Global trends in medical curriculum development emphasize the shift towards competency-based education, the integration of advanced technologies, and early clinical exposure. Competency-based education focuses on ensuring that medical graduates achieve the competencies required for effective practice, moving away from traditional time-based training models [5, 6]. The integration of technology, such as simulation-based learning and telemedicine, has revolutionized medical training by providing immersive and interactive learning experiences [7, 8]. Additionally, early clinical exposure allows students to engage with real-world healthcare settings from the beginning of their training, fostering practical skills and professional development [9, 10].

In alignment with these global shifts, the Supreme Council of Egyptian Universities (SCU) approved and implemented major reforms in the national medical education curriculum. This reform includes restructuring the existing 6-year academic program and 1-year internship into a 5-year academic phase followed by an extended 2-year internship (hence known in Egypt as “5 + 2” medical program). This reformed program emphasized competency-based education, integrated teaching and learning approaches, and the early incorporation of clinical experiences. These reforms aimed to develop versatile professionals prepared to meet the demands of contemporary healthcare. The program was aligned with the 2017 National Academic Reference Standards for Medicine (NARS-Medicine) to assist schools in designing their new curricula [11].

The new medical program was implemented in 2018, with the first cohort graduating in 2023. Recognizing the importance of assessing such a reform, the Follow-up and Support Committee, appointed by the Medical Education Sector of the SCU in Egypt, initiated an evaluation process following the graduation of the first cohort. The effectiveness of this evaluation from the perspectives of faculty and institutional leadership is essential, as understanding their viewpoints helps identify strengths, challenges, and opportunities for further improvement.

Evaluating medical education programs is crucial to ensure their quality, effectiveness, and alignment with evolving healthcare needs. Such evaluations help identify whether the curriculum meets its intended learning objectives and prepares students to address real-world challenges in healthcare [12]. Furthermore, evaluation ensures that programs remain responsive to societal and institutional demands, fostering the development of competent healthcare professionals [13].

The involvement of various stakeholders is a crucial component of program evaluation, ensuring a comprehensive assessment of its effectiveness and impact [14]. Faculty and leadership are among the key stakeholders, as their perspectives provide a comprehensive understanding of the program’s strengths and areas for improvement. The faculty members, being directly involved in teaching and mentoring, can provide insights into various aspects of the learning process, including the effectiveness of curriculum delivery, student engagement, and resource utilization [15, 16]. Leadership perspectives, on the other hand, offer a broader and more strategic view of institutional development. Their insights contribute to several key areas such as institutional goals, alignment with accreditation standards, and long-term strategic planning [17]. These perspectives ensure a balanced evaluation that addresses both operational and strategic aspects of the program.

Thus, this study aimed to evaluate the new Egyptian undergraduate medical program from the perspectives of faculty members and institutional leadership. Specifically, it explored perceptions regarding the strengths of curriculum integration, learning resources, and assessment strategies; identified key challenges encountered during program implementation; captured lessons learned throughout the implementation process; and gathered recommendations to enhance the program’s effectiveness, quality, and sustainability.

## Methods

### Study design

This study employed a concurrent (parallel) mixed-methods research design. To gain deeper insight into the findings, a cross-sectional survey was administered to faculty members, followed by focus group discussions (FGDs) with leadership from colleges of medicine.

### Study population and sample characteristics

All faculties that had implemented the new competency-based undergraduate medical curriculum in 2018, both public and private, were included in this study. A convenience, self-selection sampling method was used, where all faculty members were invited to participate. Out of 22,741, 10,728 members responded to the online survey. The response bias implications of this sampling approach are discussed in the Limitations section.

The proportion of faculty members responding from each institution ranged between 20% and 50% of their total faculty body, ensuring balanced participation. The maximum number of respondents from any single institution was 800 faculty members, representing less than 10% of the total sample (10,728 respondents). This distribution confirms that no single institution predominated in shaping the results. Furthermore, geographic representation was comprehensive, with faculties from all governorates across Egypt participating. This nationwide coverage ensured that both institutional type (public vs. private) and regional diversity were adequately represented, minimizing the risk of bias due to institutional size or geographic concentration.

A comprehensive purposive sampling strategy was used to recruit key academic leaders with direct responsibility for curriculum implementation and quality assurance across participating schools. The aim was to ensure representation of all relevant stakeholder groups rather than recruitment based solely on saturation thresholds. During analysis, recurring themes across later FGDs suggested adequate thematic saturation. Participants included deans, vice deans, program directors, and heads of quality assurance units from all 27 medical colleges nationwide. To ensure maximum variation, leaders were drawn from both public and private institutions, across different geographic regions and institutional sizes. Nine focus group discussions (FGDs) were conducted, each including representatives from 3 to 4 colleges, with 3 to 4 participants per college (total of 100 participants; 46 males and 54 females). Each FGD included 10–12 participants drawn from 3 to 4 institutions to ensure diversity of perspectives. Group size in this study exceeds the 6–8 participant range typically recommended for focus groups on the basis of maximizing individual depth of contribution. This was deliberate and driven by a primary objective of institutional representativeness: each FGD needed to include leadership from multiple colleges simultaneously so that inter-institutional dialogue (itself an analytically valuable feature of a nationally-mandated reform study) could be captured. This approach is consistent with practice in policy-evaluation research, where larger multi-stakeholder discussions are recognized as valid for surfacing collective institutional perspectives rather than purely individual experiential accounts. To mitigate the risk of dominant-voice effects, structured moderation techniques were employed, including directed questioning of all participants, round-robin responses on key items, and active facilitation of quieter voices. All invited leaders were encouraged to share both supportive and critical perspectives, and dissenting views were not excluded. This approach ensured diversity of insights and minimized the risk of selection bias. The FGDs were conducted between December 2023 and December 2024.

### Evaluation framework

We adopted the Kirkpatrick Model as the primary framework, which evaluates educational programs across four levels: reaction, learning, behavior, and results [14]. In this manuscript, our focus is on faculty and institutional leadership perspectives, which correspond mainly to the behavior (how the new curriculum influences teaching and clinical training practices) and results (perceived institutional and systemic outcomes) levels.

### Data collection

#### Quantitative data (Survey)

A structured survey form was developed by the researchers after a thorough review of existing studies and validated instruments to assess faculty perceptions of the new program across various dimensions, including program goals, curriculum content, learning resources, faculty support, and overall teaching experience. The survey was composed of 42 items, in addition to an item that explored the overall satisfaction with the teaching experience in the program. Responses were recorded on a 5-point Likert scale, ranging from 1 (Strongly Disagree) to 5 (Strongly Agree).

As the survey was a researcher-devised one, reliability and validity studies were necessary to guarantee the credibility of the collected data. The survey was tested for internal consistency through Cronbach’s alpha test. Two types of validity were established for the survey, which were content validity and construct validity. Content validity was established through revision of the survey form by seven independent reviewers, including medical educationists, public health consultants, as well as a psychometrician. Exploratory Factor Analysis (EFA) was performed to establish the construct validity of the survey.

The survey was distributed electronically through official email addresses, which each college distributed to its faculty members. Responses were collected over 3 months.

#### Qualitative data (FGDs)

Semi-structured FGDs were conducted with leadership from the colleges of medicine alongside the survey data collection. The discussions, based on an FGD guide prepared by the researchers, explored leadership’s perspectives related to the different aspects of the program (including implementation, challenges, and recommendations for improvement), providing complementary insights into the survey findings. Sessions were recorded after taking consent from all participants and transcribed by the researchers for thematic analysis.

### Data analysis

#### Exploratory factor analysis (EFA)

Principal component analysis with Varimax rotation was applied in the EFA. The decision on the number of factors to extract was guided by several criteria: the Kaiser criterion (selecting factors with eigenvalues greater than 1 [18], the Scree test (or Cattell criterion) to pinpoint the inflection point on the Scree plot [19], and the cumulative percentage of variance explained [20]. Subsequently, factor solutions were evaluated using these interpretability criteria [21]:

- A valid factor must include at least three items with loadings of 0.30 or higher.
- Items that load onto the same factor should share a common conceptual meaning.
- If an item loads on a different factor, it is considered to be measuring a different construct.

#### Quantitative data analysis

Data from the survey were analyzed using IBM SPSS v.29. Descriptive and inferential statistics were used to assess faculty perceptions and compare responses by gender and academic rank. Comparisons were made using an independent samples t-test and a one-way ANOVA test. Although Likert-scale responses are strictly ordinal in nature, the use of parametric tests (t-test and ANOVA) is well-established in educational research when the scale encompasses five or more response categories and the sample size is large, as is the case in the present study; under such conditions, parametric analyses have been shown to be robust and yield results comparable to non-parametric alternatives. Effect sizes were calculated to supplement p-value-based significance testing: Cohen’s d was computed for the gender comparison, and eta-squared (η²) for the academic rank comparison, to enable assessment of educational, as well as statistical, significance. The statistical significance level was set at *p-value* < 0.05.

#### Researcher positionality and reflexivity

Several members of the research team were directly involved in designing, implementing, or supporting the reformed Egyptian medical program at the national or institutional level. This insider positioning confers advantages (including deep contextual knowledge and access to participants) but also introduces the risk of confirmation bias, whereby findings may be unconsciously interpreted in ways that favor the reform. To mitigate this, the following procedural safeguards were adopted. First, quantitative survey data were analyzed using pre-specified statistical procedures (IBM SPSS v.29), limiting scope for subjective influence. Second, thematic analysis of the focus group discussions was conducted independently by multiple researchers prior to collective discussion, preventing any single researcher’s perspective from dominating the interpretive process. Third, disconfirming cases, critical perspectives, and implementation challenges were actively sought during analysis and are reported alongside supportive findings. Fourth, a reflexivity journal was maintained throughout the qualitative analysis to document analytical decisions, evolving interpretations, and areas of uncertainty. Dissenting views expressed by participants were retained and reported verbatim. While these measures cannot fully eliminate the influence of researcher positioning, they were designed to foster a more balanced and transparent engagement with the data. Users of these findings are encouraged to consider this positionality when drawing conclusions.

#### Qualitative data analysis

Data from the focus groups were analyzed using thematic analysis to identify key institutional perspectives and supplement the survey findings. The transcripts were systematically coded, and emerging themes were identified through an iterative process. To enhance reliability, multiple researchers independently reviewed the data, and discrepancies were resolved through discussion and consensus. Representative quotes were selected to illustrate key themes and provide deeper insight into faculty and leadership perceptions. Reporting of the quantitative component adhered to the STROBE (Strengthening the Reporting of Observational Studies in Epidemiology) checklist. Reporting of the mixed-methods component was guided by the Good Reporting of a Mixed Methods Study (GRAMMS) framework. The thematic analysis followed an inductive approach, allowing themes to emerge directly from participant data rather than being imposed by a predetermined framework [22]. The analysis was conducted manually without the use of qualitative data management software. The process proceeded through six iterative phases: familiarization with the data, generation of initial codes, searching for themes, reviewing themes, defining and naming themes, and producing the report. An audit trail was maintained throughout, documenting coding decisions and theme development at each stage to support transparency and dependability. Discrepancies between coders were resolved through discussion and consensus to reach inter-rater agreement.

## Results

This mixed-method study aimed to evaluate the new Egyptian Undergraduate Medical Program from the perspectives of faculty and institutional leadership. Using a detailed, researcher-made survey instrument that assessed various dimensions, responses were collected from a representative sample. A total of 10,728 faculty members responded to the survey, yielding a response rate of 42.2%.

The results are presented here in three sections, which are: validity and reliability studies of the survey, quantitative analysis of survey responses, and qualitative analysis of the focus group discussions.

### Section 1: Validity and reliability studies of the survey

#### Reliability study

The survey’s internal consistency was confirmed using Cronbach’s alpha test. An alpha coefficient of 0.976 was obtained, demonstrating high internal consistency. However, as alpha values exceeding 0.95 may signal item redundancy or excessive overlap between items rather than superior reliability per se, the possibility of item duplication or construct overlap warrants consideration in future refinements of this instrument.

#### Content validity

A critical review of the survey resulted in some modifications, including item rephrasing and additions. Item rephrasing examples are rephrasing item # 1 from *“The program goals and objectives were arranged to correspond with the institution’s mission and strategic objectives”* to *“Program goals and objectives were aligned with the institution’s mission and strategic objectives”*, item # 25 from *“The academic support staff were considered to have adequate knowledge about the medical curriculum”* to *“The academic support staff were knowledgeable about the medical curriculum”*, and item # 38 from *“The program offered a chance to participate in research and scholarly activities”* to *“The program provided me with several opportunities to engage in research and scholarly activities”*. Item addition examples included adding an item on the program’s suitability for preparing future doctors (item #6: *“Program content was suitable for the requirements of the future doctor”*) as well as an item on the program’s ability to suit different learning styles (item #17: *“The learning resources provided by the program suited students’ different learning styles”*).

#### Construct validity

**Fig. 1:**
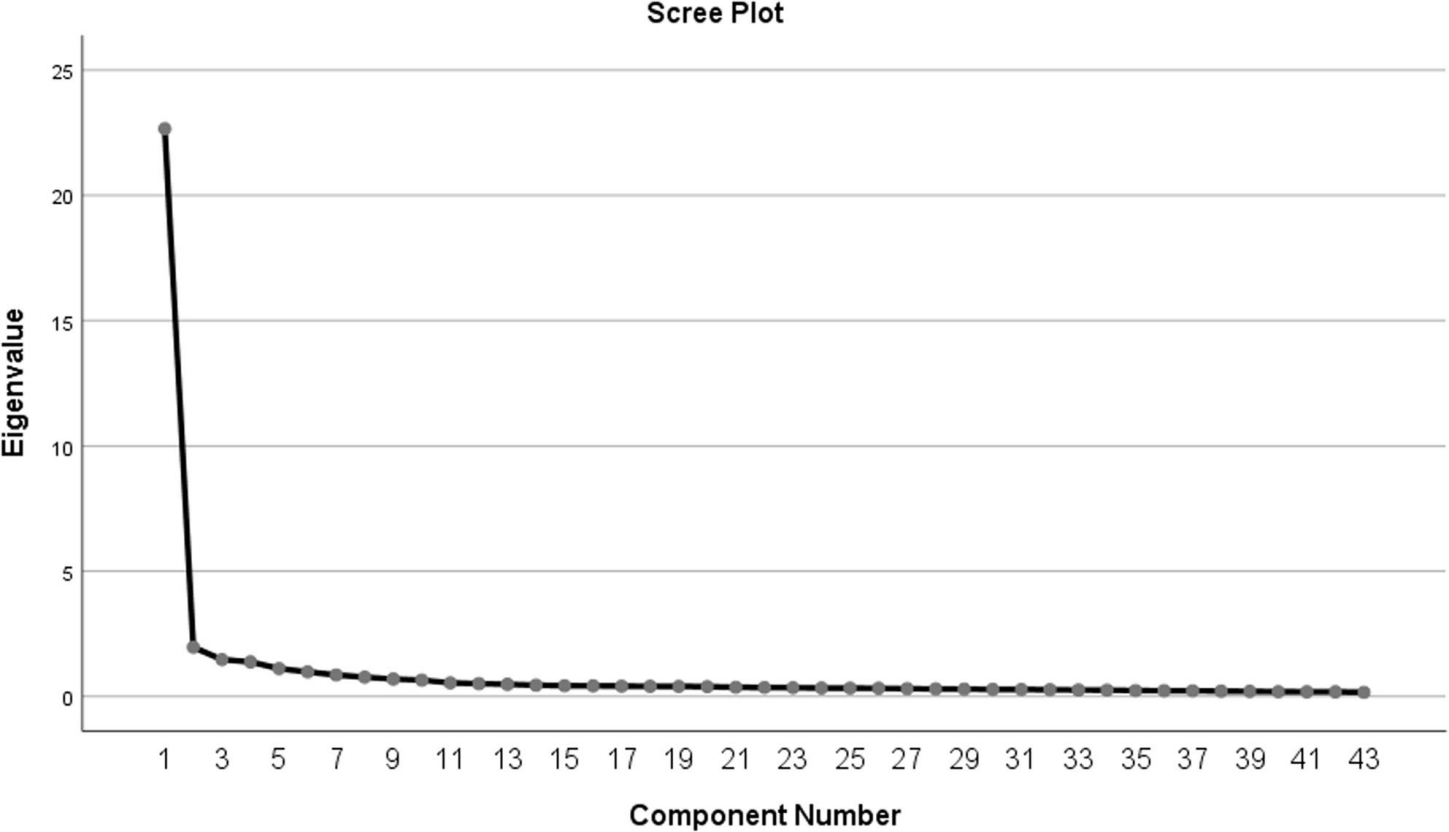
Scree plot of EFA

The construct validity was assessed using EFA (Table 1; Fig. 1). The following were found:

**Table 1:** Exploratory factor analysis (EFA)

Item No.	Item Text	Factor 1	Factor 2	Factor 3	Factor 4	Factor 5
1	Program goals and objectives were aligned with the institution’s mission and strategic objectives	0.599				
3	The students learning outcomes for the course I taught were clear	0.623				
4	The goals and objectives of the program were effectively supported through sufficient learning opportunities, including hands-on clinical experiences	0.536				
5	Program content adequately covered the fundamental knowledge, skills, and competencies required for medical practice	0.633				
6	Program content was suitable for the requirements of the future doctor	0.643				
7	Teaching methods were aligned with the learning outcomes	0.622				
8	The program encouraged using interactive teaching/learning methods (e.g., small group activities, discussion forums …)	0.453				
9	The degree of integration between the basic sciences and each other, and between them and the clinical sciences was sufficient	0.485				
10	Student assessment methods were appropriate for the learning outcomes and were in alignment with the program goals and objectives	0.636				
11	Student assessment methods were fair	0.633				
12	Student assessment methods were effective in measuring students’ knowledge, skills, and competencies	0.628				
14	The learning resources provided by the program were adequate to meet students’ and teachers’ needs	0.695				
15	The learning resources provided by the program were up to date and accurate	0.679				
16	The learning resources provided by the program were accessible and easy to use	0.711				
17	The learning resources provided by the program suited students’ different learning styles	0.659				
18	The learning resources provided by the program helped the students become independent learners	0.537				
Eigenvalue: 22.66 | Variance: 52.69% *Factor 1 Label: “Program Goals*,* Content*,* Learning Resources*,* and Student Assessment”*						
27	The program was inspiring for the students to pursue a career in medicine		0.512			
28	The program was capable of helping students develop the knowledge and skills they need to be successful doctors		0.550			
29	The program helped students to keep up to date with the latest developments in medicine		0.584			
30	The program helped students develop the ability to investigate and solve new problems		0.650			
31	The program helped students develop self-directed learning skills		0.729			
32	The program helped students develop the ability to work effectively in teams		0.730			
33	The program improved students’ communication and interpersonal skills		0.721			
34	The program helped students develop professionalism and commitment to ethical practice		0.611			
35	The program helped students develop effective basic skills in using technology in learning and research		0.660			
13	The program offered the students adequate opportunities to participate in research projects		0.511			
19	The learning opportunities offered by the program helped students develop research skills		0.627			
Eigenvalue: 1.96 | Variance: 4.56% *Factor 2 Label: “Program Impact on Students’ Professional and Research Skills”*						
36	The program encouraged me to seek external opportunities for faculty development/continuing professional development			0.697		
37	The program offered me enough faculty development/continuing professional activities			0.711		
38	The program provided me with several opportunities to engage in research and scholarly activities			0.657		
39	The program provided me with the necessary support for implementing new teaching techniques/technologies			0.655		
40	The program provided opportunities for collaboration and support among faculty members from different departments			0.606		
41	I had the opportunity to participate in curriculum improvement/reform			0.671		
42	I had the opportunity to suggest new elective courses in the new program			0.572		
2	I had the opportunity to participate in formulating the program’s mission, objectives, and learning outcomes			0.557		
Eigenvalue: 1.47 | Variance: 3.42% *Factor 3 Label: “Faculty Involvement and Support”*						
23	Academic support and career counselling services were readily available for the students				0.749	
24	Students’ academic progress was continuously monitored by their teachers				0.713	
25	The academic support staff were knowledgeable about the medical curriculum				0.786	
26	Academic support services provided by the program, in general, were satisfactory				0.762	
Eigenvalue: 1.38 | Variance: 3.20% *Factor 4 Label: “Students’ Academic Guidance and Support”*						
20	Learning facilities (i.e., lecture halls, laboratories, small group rooms …) were adequate					0.457
21	Adequate physical facilities were available for extracurricular activities (including sporting and recreational activities)					0.513
22	The facilities for clinical clerkship and practicum were useful in helping students develop their clinical skills					0.521
Eigenvalue: 1.11 | Variance: 2.58% *Factor 5 Label: “Physical and Learning Facilities”*						

- The dataset, consisting of 10,728 responses, was deemed suitable for factor analysis. The Kaiser-Meyer-Olkin (KMO) Measure of Sampling Adequacy was 0.98, and Bartlett’s Test of Sphericity was significant (χ² (903, *N* = 10728) = 147310.03, *p* < 0.001), confirming the appropriateness of the data for factor analysis.
- Factor extraction and rotation identified five factors with Eigenvalues > 1, collectively explaining 66.46% of the total variance. One item (#43: *“Overall*,* I was satisfied with my teaching experience in the program”*) was removed. Cross-loading of global satisfaction items across multiple factors is a well-recognized psychometric phenomenon, reflecting the tendency of overall appraisal items to share variance with several domain-specific constructs simultaneously. The decision to remove this item was based on its inability to contribute discriminantly to any single factor, thereby compromising the structural clarity of the instrument. The item was retained as a standalone outcome variable and analysed independently to preserve its evaluative value.
- All identified factors contained at least three items, no cross-loading was observed, and all retained items had factor loadings greater than 0.30 on their respective factors.

Following EFA, the survey retained 42 items grouped into five factors, which were named based on the items with the highest loadings and the underlying conceptual themes:

1. Factor 1 accounted for 52.69% of the variance, with an eigenvalue of 22.66. Sixteen items loaded onto this factor, with values ranging from 0.453 to 0.711. This factor was labeled *“Program Goals*,* Content*,* Learning Resources*,* and Student Assessment”*. Factor 1 encompasses conceptually diverse sub-constructs, including program goals, curriculum content, learning resources, and student assessment. While these elements loaded together statistically, this heterogeneity may reflect shared method variance or a common satisfaction orientation rather than strict construct uni-dimensionality. Furthermore, the substantial dominance of Factor 1 (52.69% of total variance), relative to the remaining four factors (collectively 13.76%), may suggest the presence of a strong general evaluative factor underlying respondents’ perceptions, which merits cautious interpretation of the five-factor solution.
2. Factor 2 explained 4.56% of the variance and had an eigenvalue of 1.96. Eleven items loaded onto it, with values between 0.511 and 0.730. This factor was labeled *“Program Impact on Students’ Professional and Research Skills”*.
3. Factor 3 explained 3.42% of the variance, with an eigenvalue of 1.47. Eight items were associated with this factor, with loadings ranging from 0.557 to 0.711. This factor was labeled *“Faculty Involvement and Support”*.
4. Factor 4 accounted for 3.20% of the variance and had an eigenvalue of 1.38. Four items loaded onto this factor, with values between 0.713 and 0.786. This factor was labeled *“Students’ Academic Guidance and Support”*.
5. Factor 5 contributed 2.58% of the variance, with an eigenvalue of 1.11. Three items were grouped under this factor, with loadings between 0.457 and 0.513. This factor was labeled *“Physical and Learning Facilities”*.

These five factors represent distinct dimensions of the survey, providing preliminary evidence of construct validity. It should be noted that, given the large sample size, confirmatory factor analysis (CFA) or split-sample cross-validation would have further strengthened these psychometric conclusions; the absence of such analyses is acknowledged as a limitation of the present validation approach. These psychometric concerns carry practical implications for interpretation. The very high alpha (α = 0.976) and the dominance of Factor 1 (52.69% of variance) together suggest that the five subscale scores may not be meaningfully distinct from one another; differences between subscales should therefore be treated as indicative rather than definitive. Scores on individual factors (particularly Factor 1, whose conceptually heterogeneous composition limits interpretive clarity) should be viewed as approximate estimates rather than precise measures of separate constructs. These concerns do not invalidate the findings, but do require that all factor-based comparisons and inferences be made with appropriate caution.

### Section 2: Quantitative analysis of survey responses

**Fig. 2:**
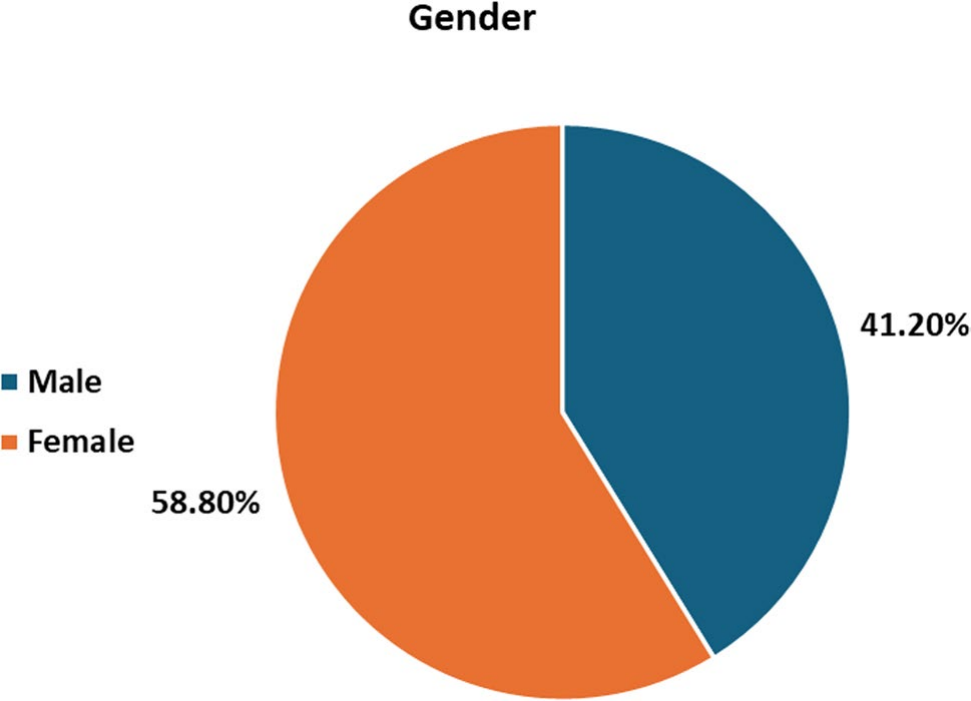
Distribution of the study participants according to gender

Figure 2 shows the gender breakdown of the study participants. Out of a total of 10,728 respondents, approximately 41% were male and 59% were female. This distribution suggests that the faculty perspectives portrayed in this study are more heavily weighted toward female respondents.

**Fig. 3:**
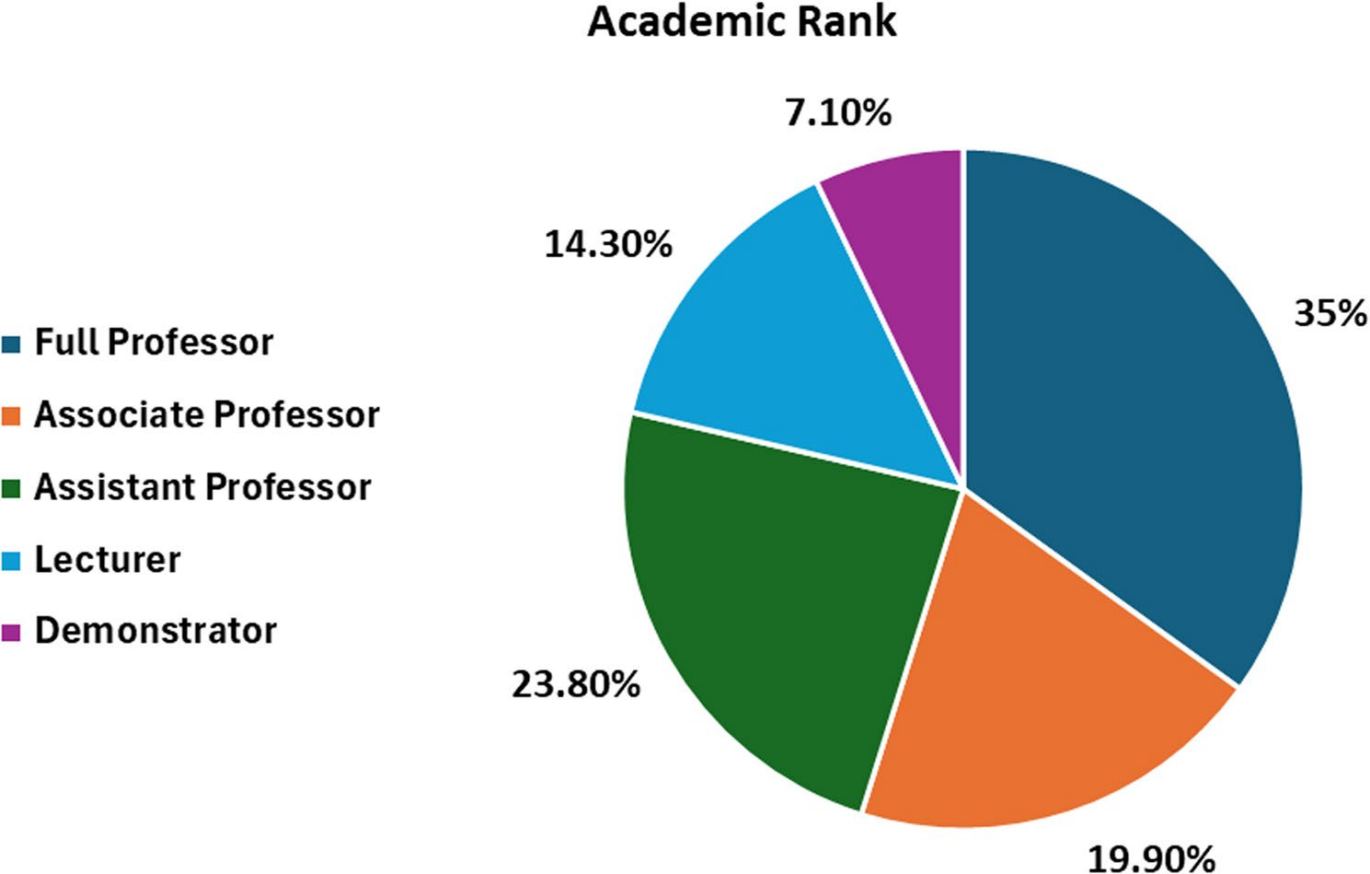
Distribution of the study participants according to academic rank

Figure 3 illustrates the academic ranks of the participants. The distribution highlights a diverse mix of academic ranks, with a higher representation of senior faculty (Full, Associate, and Assistant Professors) than junior members (Lecturers and Demonstrators).

Table 2 summarizes faculty perceptions regarding various aspects of the new undergraduate medical program. The items of the survey received mean scores that span between 2.73 and 3.61, indicating a mixed but generally moderate perception of the program’s strengths and weaknesses. Items related to learning resources—particularly their accessibility and ease of use—received higher mean scores (up to 3.61), suggesting that these are viewed favorably. On the other hand, certain items, such as the opportunity to suggest new elective courses (mean 2.86) and overall teaching experience (mean 2.99), received lower scores, indicating potential areas for enhancement.

**Table 2:** Descriptive statistics of the survey items and subscales

Item No.	Item Text	Mean (± SD)	Min – Max
Program Goals, Content, Learning Resources, and Student Assessment		3.28 (± 0.78)	
1	Program goals and objectives were aligned with the institution’s mission and strategic objectives	3.42 (± 0.98)	1–5
3	The students learning outcomes for the course I taught were clear	3.43 (± 1.03)	1–5
4	The goals and objectives of the program were effectively supported through sufficient learning opportunities, including hands-on clinical experiences	3.01 (± 1.11)	1–5
5	Program content adequately covered the fundamental knowledge, skills, and competencies required for medical practice	3.16 (± 1.07)	1–5
6	Program content was suitable for the requirements of the future doctor	3.10 (± 1.09)	1–5
7	Teaching methods were aligned with the learning outcomes	3.24 (± 0.98)	1–5
8	The program encouraged using interactive teaching/learning methods (e.g., small group activities, discussion forums …)	3.49 (± 0.99)	1–5
9	The degree of integration between the basic sciences and each other, and between them and the clinical sciences was sufficient	3.04 (± 1.05)	1–5
10	Student assessment methods were appropriate for the learning outcomes and were in alignment with the program goals and objectives	3.17 (± 1.03)	1–5
11	Student assessment methods were fair	3.48 (± 0.98)	1–5
12	Student assessment methods were effective in measuring students’ knowledge, skills, and competencies	3.19 (± 1.00)	1–5
14	The learning resources provided by the program were adequate to meet students’ and teachers’ needs	3.31 (± 0.95)	1–5
15	The learning resources provided by the program were up to date and accurate	3.44 (± 0.90)	1–5
16	The learning resources provided by the program were accessible and easy to use	3.61 (± 0.88)	1–5
17	The learning resources provided by the program suited students’ different learning styles	3.35 (± 0.91)	1–5
18	The learning resources provided by the program helped the students become independent learners	3.11 (± 0.99)	1–5
Program Impact on Students’ Professional and Research Skills		3.18 (± 0.82)	
27	The program was inspiring for the students to pursue a career in medicine	3.11 (± 1.05)	1–5
28	The program was capable of helping students develop the knowledge and skills they need to be successful doctors	3.11 (± 1.02)	1–5
29	The program helped students to keep up to date with the latest developments in medicine	3.09 (± 0.99)	1–5
30	The program helped students develop the ability to investigate and solve new problems	3.10 (± 0.99)	1–5
31	The program helped students develop self-directed learning skills	3.23 (± 0.99)	1–5
32	The program helped students develop the ability to work effectively in teams	3.27 (± 0.97)	1–5
33	The program improved students’ communication and interpersonal skills	3.31 (± 0.95)	1–5
34	The program helped students develop professionalism and commitment to ethical practice	3.13 (± 0.98)	1–5
35	The program helped students develop effective basic skills in using technology in learning and research	3.46 (± 0.92)	1–5
13	The program offered the students adequate opportunities to participate in research projects	3.03 (± 1.02)	1–5
19	The learning opportunities offered by the program helped students develop research skills	3.14 (± 0.99)	1–5
Faculty Involvement and Support		3.12 (± 0.80)	
36	The program encouraged me to seek external opportunities for faculty development/continuing professional development	3.30 (± 1.04)	1–5
37	The program offered me enough faculty development/continuing professional activities	3.10 (± 1.03)	1–5
38	The program provided me with several opportunities to engage in research and scholarly activities	2.99 (± 1.03)	1–5
39	The program provided me with the necessary support for implementing new teaching techniques/technologies	3.04 (± 1.04)	1–5
40	The program provided opportunities for collaboration and support among faculty members from different departments	3.34 (± 1.03)	1–5
41	I had the opportunity to participate in curriculum improvement/reform	3.20 (± 1.01)	1–5
42	I had the opportunity to suggest new elective courses in the new program	2.86 (± 1.00)	1–5
2	I had the opportunity to participate in formulating the program’s mission, objectives, and learning outcomes	3.09 (± 0.99)	1–5
Students’ Academic Guidance and Support		3.40 (± 0.79)	
23	Academic support and career counselling services were readily available for the students	3.49 (± 0.90)	1–5
24	Students’ academic progress was continuously monitored by their teachers	3.26 (± 0.95)	1–5
25	The academic support staff were knowledgeable about the medical curriculum	3.52 (± 0.88)	1–5
26	Academic support services provided by the program, in general, were satisfactory	3.34 (± 0.91)	1–5
Physical and Learning Facilities		2.93 (± 0.86)	
20	Learning facilities (i.e., lecture halls, laboratories, small group rooms …) were adequate	3.00 (± 1.13)	1–5
21	Adequate physical facilities were available for extracurricular activities (including sporting and recreational activities)	2.73 (± 1.01)	1–5
22	The facilities for clinical clerkship and practicum were useful in helping students develop their clinical skills	3.07 (± 0.98)	1–5
Overall:			
Overall, I was satisfied with my teaching experience in the program		2.99 (± 1.70)	1–5

The final item – “Overall, I was satisfied with my teaching experience in the program” – received a mean score of 2.99 (± 1.70). It should be noted that this mean lies marginally below the midpoint of the 5-point scale (3.0), suggesting borderline rather than clearly moderate satisfaction, and could also be interpreted as reflecting mild dissatisfaction on average. Caution is therefore warranted in characterizing this finding as “moderate” satisfaction without qualification. However, the relatively high standard deviation suggests a wide variation in perceptions among respondents. Some faculty members appear to be quite satisfied with their teaching experience, while others may have concerns or dissatisfaction.

Table 3 compares overall teaching experience scores by gender and academic rank. Male and female respondents reported identical mean scores (2.99), with no statistically significant difference (*p* = 0.955). The effect size was trivial (Cohen’s d ≈ 0.00), confirming the absence of any meaningful gender-based difference. This suggests that overall teaching experience is perceived similarly across genders. There is a statistically significant difference in overall teaching experience scores among academic ranks (*p* < 0.001). Full Professors, Associate Professors, and Assistant Professors reported mean scores ranging from 2.90 to 2.98, whereas Lecturers and Demonstrators reported higher mean scores (3.18 and 3.16, respectively). The effect size for academic rank was small (eta-squared, η² ≈ 0.008), indicating that, despite the statistically significant difference attributable to the very large sample size, academic rank accounts for less than 1% of the variance in overall teaching experience scores. This suggests that the practical (educational) significance of rank-based differences is limited. This indicates that faculty in lower academic ranks perceive their teaching experience more positively than those in higher ranks.

**Table 3:** Comparison of overall teaching experience in the program among different groups of study participants

Characteristic	Mean (± SD)		*p*-value	Effect Size
Gender				
Male *(n = 4421)*	2.99 (± 1.21)		0.955	Cohen’s d ≈ 0.00 (trivial effect)
Female *(n = 6307)*	2.99 (± 1.14)			
Academic Rank^§^				
Full Professor *(n = 3755)*	2.90 (± 1.19)		< 0.001*	η² ≈ 0.008 (small effect)
Associate Professor *(n = 2132)*	2.98 (± 1.18)			
Assistant Professor *(n = 2549)*	2.95 (± 1.15)			
Lecturer *(n = 1531)*	3.18 (± 1.12)			
Demonstrator *(n = 761)*	3.16 (± 1.08)			

*Statistically significant

^§^The rank comparison is unadjusted and assumes independence of observations

### Section 3: Qualitative analysis of focus group discussions

Nine focus groups were conducted to explore institutional leaders’ perspectives on the new Egyptian medical curriculum. Each focus group included leadership from three to four medical schools. The number of participants in each FGD ranged from 10 to 15 (Deans, Vice Deans for Educational Affairs, Program Directors, and Quality Unit Directors) and each FGD extended over 45 to 60 min. The total number of participants in all FGDs was 100, 46% were males and 54% were females. In terms of academic rank, 75% were professors, while 25% were associate professors. Regarding their administrative roles, 25% were Deans, 27% were Vice Deans, 22% were Program Directors, and 26% were Quality Assurance Unit Directors. Four themes emerged from the focus group discussions: Teaching and Learning Strategies in the 5 + 2 Program, Challenges in Program Implementation, Student Experience and Feedback, and Leadership and Institutional Support. The thematic analysis of the qualitative data is presented in Table 4.

**Table 4:** Thematic analysis of the FGDs with institutional leadership *(n = 100)*

Themes	Sub-themes	Codes
Teaching and Learning Strategies in the 5 + 2 Program	Integration between Basic and Clinical Sciences	- Spiral, horizontal, and vertical integration - Reduced conflicts and gaps between disciplines - Collaboration across departments for better connections
	Early Clinical Exposure	- Enhanced preparedness for clinical phases - Development of basic clinical and procedural skills through skills labs and field training - Increased enthusiasm among pre-clinical students
	Modern Teaching and Assessment Methods	- Use of interactive methods (PBL, CBL, flipped classrooms) - Implementation of evidence-based medicine and research - Diversification of assessment tools (OSCE, formative assessments)
Challenges in Program Implementation	*Facilities* and Infrastructure Issues	- Insufficient faculty members and training spaces - Overcrowding due to increased student intake - Limited availability of some equipment in hospitals and labs
	Resistance to Change and Faculty Development	- Reluctance of some senior faculty to adopt new teaching methodologies - Limited experience in competency-based learning approaches - Need for continuous faculty training in modern education techniques
	Assessment and Curriculum Design	- Traditional exam formats persist - High student workload due to heavy content - Variability in grading and module content across colleges
Student Experience and Feedback	Student Challenges	- Overloaded curriculum and frequent assessments - Inconsistencies in teaching across colleges - Need for better scheduling and workload management
	Student Empowerment and Engagement	- Inclusion of student feedback on curriculum improvement - Formation of student support committees - Encouragement of research and peer collaboration
	Feedback Mechanisms and Adjustments	- Regular surveys - Direct communication with module coordinators to convey opinions - Modifications in course content and examination formats based on student feedback - Creation of formative, case-based learning questions based on student feedback
Leadership and Institutional Support	University Leadership’s Role	- Provision of infrastructure, digital platforms, and funding whenever possible - Expansion of electronic exam labs and e-learning initiatives - Introduction of student-centered learning strategies
	Faculty Development and Training Programs	- Workshops on teaching methodologies and curriculum design - Training at national and international institutions - Integration of training attendance into faculty promotions
	Sustainability and Continuous Development	- Regular review of modules and program outcomes - Standardization of educational policies across colleges - Ensuring alignment with competency-based learning goals

### Theme 1. Teaching and learning strategies in the 5 + 2 program

#### Integration between basic and clinical sciences

The findings indicate that integration of basic and clinical sciences has been well-received, with many participants acknowledged its positive impact on reducing knowledge gaps and enhancing collaboration.

One participant stated, *“Collaboration across departments for integration has significantly improved our understanding*,* reduced redundancy*,* and fostered better connections.”*

This integration, as stated, has also helped eliminate repetition in the curriculum, leading to a more streamlined and cohesive learning experience.

#### Early clinical exposure

Early clinical exposure was widely regarded as beneficial for students, with several participants reported that it enhanced students’ confidence when transitioning to clinical rotations.

One participant stated, *“Early exposure makes students more confident when transitioning to clinical rotations.”*

This early engagement with real-world clinical settings helped students feel more prepared and comfortable in applying their knowledge to practice.

#### Modern teaching and assessment methods

The integration of interactive, student-centered, and modern teaching methods, such as problem-based learning (PBL), case-based learning (CBL), and flipped classrooms (FC), was praised by several participants for making learning more engaging. However, some participants emphasized the need for improved faculty training to enhance the effectiveness of these approaches. Additionally, the incorporation of evidence-based medicine, research skills, and elective courses was highlighted as a valuable addition to the curriculum.

One participant stated, *“The new teaching strategies have made learning more engaging and applicable to real-life scenarios.”*

There was a general consensus among the focus group participants that the diversification of assessment methods in the new program, including Objective Structured Clinical Examinations (OSCE), Objective Structured Practical Examinations (OSPE), portfolios, and formative assessments, was a highly positive and commendable feature.

### Theme 2. Challenges in 5 + 2 program implementation

#### Facilities and infrastructure issues

A few participants reported facing challenges related to facility shortages, which acted as a barrier to effective practical learning. One participant stated, *“We struggle with a lack of some resources*,* making it difficult to practice hands-on skills effectively.”*

This underscores the need to ensure adequate resources are available in certain colleges.

#### Resistance to change and faculty development

Resistance to change among faculty members was another challenge, with some participants noted reluctance to adopt new teaching methodologies. One participant stated, *“Some faculty members are still hesitant about adopting new teaching styles*,* which slows down progress.”*

Additionally, there was resistance, particularly from some senior faculty, regarding changes such as reducing program duration or decreasing teaching hours for their respective specialties. This reluctance highlights the need for faculty development initiatives and clear communication on the benefits of curriculum reforms.

#### Assessment and curriculum design

Several participants highlighted concerns regarding assessment and curriculum design, emphasizing the need for a more balanced structure and improved alignment of assessment with integrated learning. Key challenges included the persistence of certain traditional exam formats (despite the introduction of new authentic methods), high student workload due to heavy content, and variability in grading and module content across colleges. One participant stated, *“The curriculum is overloaded*,* and assessment methods need better alignment with integrated learning.”*

### Theme 3. Student experience and feedback

#### Student challenges

During the early stages of program implementation, student feedback was initially negative, as many struggled to adapt to the integration strategy. However, perceptions improved as the program progressed. Key challenges included curriculum overload with frequent assessments, inconsistencies in teaching across different colleges, and the need for better scheduling and workload management. One participant stated, *“It’s difficult to balance all subjects given the heavy content and frequent exams.”*

These findings highlight the importance of regular revision of some modules to avoid redundancy and overload.

#### Student empowerment and engagement

Students were given significant opportunities to voice their perspectives, with a strong emphasis on empowering student feedback for curriculum improvement, fostering research engagement, and encouraging peer collaboration. One of the participants highlighted that *“Having a platform for student feedback has helped bring about meaningful curriculum adjustments.”*

Additionally, student support committees were established in some colleges to address all academic and psychological concerns, ensuring a more student-centered approach to curriculum development.

#### Feedback mechanisms and adjustments

Feedback mechanisms, such as surveys and direct communication with coordinators, played a crucial role in addressing student concerns. One participant stated, *“Surveys and direct communication have helped improve exam structures and learning material.”*

Examples from the feedback included students appreciating early clinical exposure and skill labs but also expressing concerns about overloaded modules and exam schedules. In response, formative case-based questions were introduced, heavy modules were divided into smaller sections, and examination schedules were adjusted to better align with student preferences. Positive feedback highlighted the program’s relevance to the labor market and improved performance during internships, demonstrating the effectiveness of the adjustments made.

### Theme 4. Leadership and institutional support

#### University leadership’s role

The feedback suggests that while the university leadership has shown support in terms of providing infrastructure, digital platforms, and funding, there is still room for improvement. Expansion of electronic exam labs and e-learning initiatives have been beneficial, but some participants believe that more funding and enhanced digital infrastructure are needed for further progress. One participant stated, *“The leadership has been supportive*,* but more needs to be done in terms of funding and digital infrastructure.”*

This indicates that while leadership efforts are acknowledged, additional investment in digital resources and financial support is essential to fully realize the potential of the institution’s academic and technological advancements.

#### Faculty development and training programs

Faculty development and training programs, including workshops on teaching methodologies, curriculum design, and training at national and international institutions, have been beneficial. Additionally, integrating the attendance of faculty training activities into faculty promotions has helped enhance teaching quality. However, despite these efforts, some faculty members still face challenges in adapting to modern teaching approaches. One participant stated, *“Faculty training programs have helped*,* but some faculty still struggle to adapt to modern approaches.”*

This suggests that while training initiatives have made a positive impact, there may still be resistance or difficulty in fully embracing new methodologies, and further support may be needed to ensure all faculty members are equipped to implement modern teaching strategies effectively.

#### Sustainability and continuous development

The participants of different focus groups highlighted the importance of regularly reviewing the educational modules and program outcomes to ensure that the curriculum remains up-to-date and aligns with the best practices. The attempts to standardize educational policies across colleges and the effort to ensure alignment with competency-based learning goals reflect a commitment to maintaining high-quality education. One of the participants emphasized, *“Ongoing review and refinement of modules ensure we stay aligned with educational best practices.”*

This suggests that continuous improvement and regular updates to the curriculum are seen as essential for ensuring that the program meets current educational standards and effectively prepares students for future challenges. However, the feedback also implies that the process of review and refinement must remain consistent to guarantee long-term sustainability and relevance.

## Discussion

This study utilized a concurrent mixed-methods design, integrating a nationwide cross-sectional survey of 10,728 faculty from Egyptian medical colleges with nine FGDs that included 100 academic leaders. The purpose was to investigate the perceptions of faculty and leadership regarding the newly implemented reformed national medical program. A structured survey composed of 42 Likert-scale items evaluated faculty satisfaction, while thematic analysis of the FGDs provided more in-depth insights into the program’s implementation, its challenges, and opportunities for enhancement. It is important to note that the findings of this study reflect the perceptions of faculty and institutional leadership rather than objective measures of educational effectiveness. Causal inferences cannot be drawn from this cross-sectional, perception-based design.

The survey results indicated a borderline to moderate level of satisfaction among faculty, with their responses yielding average mean scores across various items. A particularly noteworthy highlight was the strong appreciation for learning resources, which were praised for their accessibility, user-friendliness, and adaptability to cater to various learning preferences. This positive feedback is consistent with prior research that underscores the critical role of quality educational materials in fostering student engagement and supporting self-directed learning [23].

The evaluation of student assessment methods by faculty members received a highly positive response, particularly in terms of fairness and their alignment with educational objectives. The incorporation of varied assessment techniques, like Objective Structured Clinical Examinations (OSCEs) and portfolios, aligns with competency-based approaches and reflects established best practices in global medical education [24, 25]. However, there was a notable discrepancy in satisfaction levels among faculty, particularly when comparing academic ranks. Senior faculty members (full, associate, and assistant professors) expressed lower satisfaction than lecturers and demonstrators, which may be attributed to the increased administrative responsibilities and workload of senior faculty in the new program. While increased administrative responsibilities and workload among senior faculty offer one plausible explanation for the observed satisfaction gap, other interpretations warrant consideration. Senior faculty may experience greater resistance to pedagogical reform due to long-established disciplinary identities and deeply ingrained teaching practices that are difficult to alter [26]. Reduced curricular control, particularly the loss of autonomy over content scope and hours allocated to specific specialties may also contribute to dissatisfaction among those with longer tenure in traditional teaching roles. Additionally, administrative burden associated with accreditation, quality assurance, and curriculum governance may disproportionately affect senior academic members, further limiting their capacity to engage positively with the reform. Future studies should explore these dimensions through targeted qualitative inquiry with senior faculty. This observation also resonates with previous research on the impact of institutional support, faculty workload, and engagement in teaching innovation [27, 29]. Bridging this gap may necessitate customized professional development initiatives, mentorship programs, and a more inclusive approach to teaching innovation and participation in decision-making. The faculty expressed notable dissatisfaction regarding opportunities for engaging in research and contributing to the design of elective courses, highlighting issues of constrained academic autonomy. This concern aligns with findings from a study at Shiraz University of Medical Sciences, where faculty members identified a lack of financial independence and reliance on governmental budgets as significant factors limiting academic freedom and institutional autonomy [30]. Additionally, participants expressed significant concerns about the availability and adequacy of physical and learning infrastructure for extracurricular activities, including sports and recreational tools, which garnered the lowest satisfaction ratings. This echoes existing literature that underscores the importance of well-equipped educational spaces in enhancing the quality of medical education [31].

Gender-based analysis revealed no statistically significant differences in satisfaction regarding teaching experience in the newly implemented medical curriculum. This could reflect a fair and well-implemented system where gender did not significantly influence how educators experienced their teaching roles. This finding is supported by the findings of a study by Sadeghi et al. [32], who found that teacher job satisfaction levels are independent of gender. Conversely, studies by Sharkas et al. [33] and Okpara et al. [34] contrast this finding, reporting that female educators were significantly more satisfied with their teaching experiences in integrated curricula. They attributed this increased satisfaction among female educators to factors such as a more collaborative work environment and greater support for work-life balance within integrated curricula.

Overall satisfaction with the teaching experience was borderline to moderate, yet highly variable across respondents. This variation echoes global findings from studies in both European and U.S. contexts [28, 35], which emphasize the critical role of workload balance, professional development, and institutional support in determining faculty satisfaction. Additionally, the fact that the program was newly implemented may have contributed to the observed borderline satisfaction level; it is worth reiterating that the overall mean of 2.99 falls marginally below the scale midpoint, meaning these findings are consistent with a mild level of dissatisfaction as much as with moderate satisfaction.

Qualitative insights gathered from FGDs added significant depth to the findings. Academic leaders perceived that the integration of basic and clinical sciences enhanced collaboration and minimized redundant content, which aligns with the educational frameworks that promote curriculum integration as described by [36]. The participants also positively reported that early clinical exposure contributed to students’ perceived confidence and motivation, which aligns with two similar studies by Dahle et al. [37] and Prince et al. [38]. The shift toward student-centered learning strategies, like PBL, CBL, and FC, was viewed as a constructive evolution that makes learning more interactive and relevant. Nonetheless, the effective implementation of these methods largely relies on faculty readiness, underscoring the necessity for comprehensive faculty training programs [28].

While a variety of student assessment methods received general support, participants identified practical challenges such as increased faculty workload and issues related to standardization, mainly because of the increased frequency of assessments and the introduction of new assessment methods. Achieving consistency, fairness, and sustainability in assessment processes will require strong institutional frameworks and ongoing faculty training [39].

Areas for improvement include faculty adaptation, as many educators require further training to implement these new teaching methods effectively. In some colleges, resource limitations and infrastructure constraints also pose challenges in ensuring high-quality clinical training environments. Addressing these challenges will be critical to maximizing the program’s impact and enhancing the quality of medical education in Egypt [11]. Furthermore, while the program aligns with international medical education standards, continuous faculty development and institutional support are necessary to sustain long-term success. This study highlights a complex landscape of faculty perceptions regarding the newly implemented reformed medical program. Although there are notable strengths, particularly in learning resource availability, assessment methods, and curriculum integration, some challenges persist. These include limited research opportunities, infrastructural inadequacies, and a pressing need for continuous faculty development. Addressing these multifaceted issues is vital for enhancing faculty satisfaction and improving the quality and effectiveness of medical education in Egypt. Focusing on equity, engagement, and empowerment across all faculty levels, along with ongoing support from the institutions, can help create a more inclusive and vibrant academic environment that benefits educators and students.

Although this study shows the strengths of employing a mixed-method design and a large sample size, it still has some limitations. First, the study relied on convenience, self-selection sampling and self-reported survey responses, introducing interrelated forms of response bias. Faculty with strong views — whether as motivated reform advocates or those with pronounced dissatisfaction — may have been disproportionately inclined to respond, while those with neutral or moderately positive perceptions may be under-represented. Self-reported responses may also reflect institutional expectations rather than objective assessments, and faculty overburdened with administrative tasks may have been less likely to participate. Importantly, a large response number (*n* = 10,728) does not, by itself, eliminate these risks; a systematically biased large sample may still misrepresent the population’s true views. Second, while the program is nationally standardized, differences in implementation fidelity across the 27 medical schools (e.g., variations in resources, faculty training, infrastructure …) could have influenced faculty experiences. Third, this study focused solely on the perceptions of faculty and institutional leadership. Student perspectives, which are central in competency-based education, given that students are the primary beneficiaries of the curriculum were not incorporated in this study. However, a separate companion study exploring the perspectives and academic experiences of the first graduating cohort has been conducted by the team and is currently under review; together, these studies offer a more comprehensive evaluation of the program’s impact. Furthermore, no objective outcome data such as graduate competency assessments, national licensing examination performance, or internship evaluations were examined. Future research should incorporate such measures to enable a more complete appraisal of the program’s educational effectiveness. Fourth, three interrelated analytical limitations warrant acknowledgment. The psychometric validation of the survey relied exclusively on exploratory factor analysis (EFA), without subsequent confirmatory factor analysis (CFA) or split-sample cross-validation; these would have strengthened the evidence base for construct validity. Additionally, the one-way ANOVA comparison by academic rank did not control for confounding variables that co-vary with rank, including age, years of teaching experience, administrative workload, and leadership role; multivariable regression would have permitted more refined attribution of the observed differences. Finally, the nested structure of the data (faculty embedded within 27 institutions) was not accounted for statistically, potentially violating the assumption of independence and resulting in underestimated standard errors and overly optimistic p-values. Readers should therefore interpret all significance levels in the quantitative comparisons with this caveat in mind. Multilevel or hierarchical modelling, which would properly partition variance between the institutional and individual levels, is the methodologically appropriate approach and is strongly recommended for future work.

## Conclusion

This study provides a comprehensive examination of faculty and academic leadership perceptions regarding the newly implemented reformed national medical program in Egypt. The findings reveal a landscape marked by both commendable aspects, persistent challenges, and areas for improvement. Faculty satisfaction was generally moderate, with high appreciation expressed for learning resources, assessment methods, integration of basic and clinical sciences, and early clinical exposure—hallmarks of a modern, student-centered medical curriculum. However, disparities in satisfaction levels based on academic rank, along with concerns about limited research opportunities, insufficient infrastructure in some colleges, and increased faculty workload, highlight areas that require attention and targeted intervention. It should be emphasized that these findings represent stakeholder perceptions and do not constitute evidence of educational effectiveness in terms of graduate competencies, licensing outcomes, or clinical performance. The findings of this study may provide valuable insights for policymakers and educators involved in similarly centralized, competency-based curriculum reform initiatives, particularly in contexts characterized by comparable national governance structures.

## Supplementary Information

Supplementary Material 1.

Supplementary Material 2.

## Data Availability

Data is available from the corresponding author upon reasonable request.

## Abbreviations

5 + 2 Program: Five-Year Academic Phase + Two-Year Internship
CBL: Case-Based Learning
CFA: Confirmatory Factor Analysis
EFA: Exploratory Factor Analysis
FC: Flipped Classroom
FGD: Focus Group Discussion
FGDs: Focus Group Discussions
KMO: Kaiser-Meyer-Olkin Measure of Sampling Adequacy
NARS: National Academic Reference Standards
OSCE: Objective Structured Clinical Examination
OSPE: Objective Structured Practical Examination
PBL: Problem-Based Learning
SCU: Supreme Council of Egyptian Universities
SPSS: Statistical Package for the Social Sciences
χ²: Chi-Squared Test
η²: Eta-squared (effect size measure for one-way analysis of variance)
