# Supplementary material for: Evaluation of the new Egyptian undergraduate medical program from the perspectives of faculty and institutional leadership: a nationwide, mixed-method study
Source: BMC Med Educ. 2026 Aug 13;26:1311. doi: 10.1186/s12909-026-09865-6 (PMC13471601; doi:10.1186/s12909-026-09865-6)
Supplement: Supplementary file 1 [file 12909_2026_9865_MOESM1_ESM.docx]

New 5+2 Medicine Program Evaluation

*Faculty and Assistant Staff Survey*

Name of Medical School & University: ………………………

Type of Medical School: [	] Governmental	[	] Public	[	] Private	[	] Military

*Dear Faculty and Assistant Staff of the 5+2 Program,*

The purpose of this survey is to evaluate the “5+2 Bachelor of Medicine and Surgery Program” from your point of view. Your feedback is important in this regard, and it will be used to improve the program for the future. The survey is anonymous and confidential.

Please choose the answer that is closest to your personal experience as much as possible, based on the courses you have taught.

Thank you for your time and effort.

Part 1: Demographic Data:

**Name *(optional)*:** ………………………….	**Age:** ………………………….

Gender: [	] Male	[	] Female	Years of Teaching Experience: ………………………….

Specialty/Department: ………………………….………………………….

Academic Rank:	[	] Full Professor	[	] Assistant Professor	[	] Lecturer	[	] Assistant Lecturer	[	] Demonstrator

Part 2:

Direction: For each of the statements in the table below, choose the option that represents your opinion, where:

- Strongly Agree (SA): You are in full agreement with the statement and believe it to be totally true.
- Agree (A): You agree with the statement and believe it to be mostly true.
- Sometimes (S): You believe the statement to be true some of the time, but not always.
- Disagree (D): You do not agree with the statement and believe it to be mostly false.
- Strongly Disagree (SD): You completely oppose the statement and believe it to be totally false.
- Not Applicable (NA): The statement does not apply to your case/you don’t know or are not sure.

| **No.** | **Statement** | **SA** | **A** | **S** | **D** | **SD** | **NA** |
| --- | --- | --- | --- | --- | --- | --- | --- |
| **Section 1: Program Goals and Objectives, Content, and Learning Opportunities:** | | | | | | | |
| 1 | Program goals and objectives were aligned with the institution’s mission and strategic objectives |  |  |  |  |  |  |
| 2 | I was involved in formulating the program's mission, objectives, learning outcomes, and course contents of the program |  |  |  |  |  |  |
| 3 | The student learning outcomes for the course I taught were clear |  |  |  |  |  |  |
| 4 | The program offered the students enough learning opportunities (including hands-on clinical experiences) to achieve its goals and objectives |  |  |  |  |  |  |
| 5 | Program content adequately covered the fundamental knowledge, skills, and competencies required for medical practice |  |  |  |  |  |  |
| 6 | Program content was suitable for the requirements of the future doctor |  |  |  |  |  |  |
| 7 | Teaching methods were aligned with the learning outcomes |  |  |  |  |  |  |
| 8 | The program encouraged using interactive teaching/learning methods (e.g., small group activities, discussion forums …) |  |  |  |  |  |  |
| 9 | The degree of integration between the basic sciences and each other, and between them and the clinical sciences was sufficient |  |  |  |  |  |  |
| 10 | Student assessment methods were appropriate for the learning outcomes and in alignment with the program goals and objectives |  |  |  |  |  |  |
| 11 | Student assessment methods were fair |  |  |  |  |  |  |
| 12 | Student assessment methods were effective in measuring students' knowledge, skills, and competencies |  |  |  |  |  |  |
| 13 | The program offered the students adequate opportunities to participate in research projects |  |  |  |  |  |  |
| **If you have additional comments related to this section of the questionnaire, please add them here:**  …………………………………………………………………………………………………………………………………………………  …………………………………………………………………………………………………………………………………………………  ………………………………………………………………………………………………………………………………………………… | | | | | | | |
| **Section 2: Learning Resources:** | | | | | | | |
| 14 | The learning resources provided by the program were adequate to meet students’ and teachers’ needs |  |  |  |  |  |  |
| 15 | The learning resources provided by the program were up-to-date and accurate |  |  |  |  |  |  |
| 16 | The learning resources provided by the program were accessible and easy to use |  |  |  |  |  |  |
| 17 | The learning resources provided by the program suited student’s different learning styles |  |  |  |  |  |  |
| 18 | The learning resources provided by the program helped the students become independent learners |  |  |  |  |  |  |
| 19 | The learning resources provided by the program helped students develop research skills |  |  |  |  |  |  |
| 20 | Physical facilities (i.e., lecture halls, laboratories, small group rooms …) were adequate |  |  |  |  |  |  |
| 21 | Adequate facilities were available for extracurricular activities (including sporting and recreational activities) |  |  |  |  |  |  |
| 22 | The clinical clerkship, practicum, and cooperative training programs were effective in developing students' clinical skills |  |  |  |  |  |  |
| **If you have additional comments related to this section of the questionnaire, please add them here:**  …………………………………………………………………………………………………………………………………………………  …………………………………………………………………………………………………………………………………………………  ………………………………………………………………………………………………………………………………………………… | | | | | | | |
| **Section 3: Students’ Academic Guidance and Support:** | | | | | | | |
| 23 | Academic support and career counselling services were readily available for the students |  |  |  |  |  |  |
| 24 | Students' academic progress was continuously monitored by their teachers |  |  |  |  |  |  |
| 25 | The academic support staff were knowledgeable about the medical curriculum |  |  |  |  |  |  |
| 26 | Overall, academic support services provided by the program were satisfactory |  |  |  |  |  |  |
| **If you have additional comments related to this section of the questionnaire, please add them here:**  …………………………………………………………………………………………………………………………………………………  …………………………………………………………………………………………………………………………………………………  ………………………………………………………………………………………………………………………………………………… | | | | | | | |
| **Section 4: Program Impact on Students:** | | | | | | | |
| 27 | The program was inspiring to the students to pursue a career in medicine |  |  |  |  |  |  |
| 28 | The program was capable to help students develop the knowledge and skills they need to be successful doctors |  |  |  |  |  |  |
| 29 | The program helped students to keep up-to-date with the latest developments in medicine |  |  |  |  |  |  |
| 30 | The program helped students develop the ability to investigate and solve new problems |  |  |  |  |  |  |
| 31 | The program helped students develop self-directed learning skills |  |  |  |  |  |  |
| 32 | The program helped students develop the ability to work effectively in teams |  |  |  |  |  |  |
| 33 | The program improved students’ communication and interpersonal skills |  |  |  |  |  |  |
| 34 | The program helped students develop professionalism and commitment to ethical practice |  |  |  |  |  |  |
| 35 | The program helped students develop good basic skills in using technology in learning and research |  |  |  |  |  |  |
| **If you have additional comments related to this section of the questionnaire, please add them here:**  …………………………………………………………………………………………………………………………………………………  …………………………………………………………………………………………………………………………………………………  ………………………………………………………………………………………………………………………………………………… | | | | | | | |
| **Section 5: Faculty Support:** | | | | | | | |
| 36 | The program encouraged me to seek external opportunities for faculty development/continuing professional development |  |  |  |  |  |  |
| 37 | The program offered me enough faculty development/continuing professional activities |  |  |  |  |  |  |
| 38 | The program provided me with different opportunities to engage in research and scholarly activities |  |  |  |  |  |  |
| 39 | The program provided me the necessary support for implementing new teaching techniques/technologies |  |  |  |  |  |  |
| 40 | The program provided opportunities for collaboration and support among faculty members from different departments |  |  |  |  |  |  |
| **If you have additional comments related to this section of the questionnaire, please add them here:**  …………………………………………………………………………………………………………………………………………………  …………………………………………………………………………………………………………………………………………………  ………………………………………………………………………………………………………………………………………………… | | | | | | | |
| **Section 6: Overall Experience:** | | | | | | | |
| 41 | I had the opportunity to participate in curriculum improvement/reform |  |  |  |  |  |  |
| 42 | Students were given the opportunity to participate in curriculum improvement/reform |  |  |  |  |  |  |
| 43 | Overall, I was satisfied with my teaching experience in the program |  |  |  |  |  |  |
| **If you have additional comments related to this section of the questionnaire, please add them here:**  …………………………………………………………………………………………………………………………………………………  …………………………………………………………………………………………………………………………………………………  ………………………………………………………………………………………………………………………………………………… | | | | | | | |
| **Open-ended Questions:** | | | | | | | |
| 1. **Through your work in the old curriculum (6+1) and the new curriculum (5+2), mention 2 to 3 differences between them (either negative or positive):**   …………………………………………………………………………………………………………………………………………………….  …………………………………………………………………………………………………………………………………………………….  ……………………………………………………………………………………………………………………………………………………. | | | | | | | |
| 2. **What did you like most about the program?**   ……………………………………………………………………………………………………………………………………………………………………………………………………………………………………………………………………………………………… | | | | | | | |
| 3. **What did you dislike most about the program?**   ……………………………………………………………………………………………………………………………………………………………………………………………………………………………………………………………………………………………… | | | | | | | |
| 4. **What were the challenges/obstacles you faced during implementing the program?**   …………………………………………………………………………………………………………………………………………………………………………………………………………………………………………………………………………………………… | | | | | | | |
| 5. **What suggestions do you have for improving the program?**   ……………………………………………………………………………………………………………………………………………………………………………………………………………………………………………………………………………………………… | | | | | | | |

*Thank you …*
