# Supplementary material for: Evaluation of the new Egyptian undergraduate medical program from the perspectives of faculty and institutional leadership: a nationwide, mixed-method study
Source: BMC Med Educ. 2026 Aug 13;26:1311. doi: 10.1186/s12909-026-09865-6 (PMC13471601; doi:10.1186/s12909-026-09865-6)
Supplement: Supplementary file 2 [file 12909_2026_9865_MOESM2_ESM.docx]

**Reporting Checklists**

*Manuscript: Evaluation of the New Egyptian Undergraduate Medical Program from the Perspectives of Faculty and Institutional Leadership: A Nationwide, Mixed-Method Study*

Journal: BMC Medical Education

*This supplementary file contains two complementary reporting checklists: (1) GRAMMS for the mixed-methods design and (2) COREQ for the qualitative focus group component. Together they provide comprehensive reporting transparency across both study strands. The quantitative component follows the STROBE checklist, as stated in the manuscript Methods section.*

| **GRAMMS — Good Reporting of A Mixed Methods Study (O'Cathain et al., 2008, J Clin Epidemiol)** | | | |
| --- | --- | --- | --- |
| **No.** | **GRAMMS Criterion** | **Where Addressed in Manuscript** | **Page** |
| **1** | **Describe the justification for using a mixed-methods approach to the research question.** | Stated in Study Design section: a concurrent (parallel) mixed-methods design was chosen to integrate quantitative faculty survey data with qualitative FGD insights, enabling triangulation of perceptions across faculty and leadership. | p. 6 |
| **2** | **Describe the design in terms of the purpose, priority, and sequence of methods.** | Concurrent parallel design described in Study Design section. Both quantitative (survey) and qualitative (FGDs) components were of equal priority and conducted simultaneously; integration occurred at the interpretation stage. | p. 6 |
| **3** | **Describe each method in terms of sampling, data collection, and analysis.** | Quantitative: Study Population section (sampling), Data Collection section (online survey, 3 months), Data Analysis section (SPSS v.29, EFA, t-test, ANOVA). Qualitative: Study Population section (purposive sampling, 9 FGDs, 100 leaders), Data Collection section (semi-structured FGDs), Qualitative Data Analysis section (thematic analysis, 6 phases, manual coding). | pp. 6–11 |
| **4** | **Describe where integration has occurred, how it has occurred, and who has participated in it.** | Integration is described in the Discussion section, where quantitative satisfaction findings are interpreted alongside qualitative themes (e.g., learning resources, faculty resistance, assessment challenges). Integration was achieved through narrative synthesis by the full research team. | pp. 23–27 |
| **5** | **Describe any limitation of one method associated with the presence of the other method.** | Discussed in Limitations section: self-selection sampling in the survey may not fully represent faculty with neutral views; the qualitative FGD component involved non-standard large group sizes (10–12 per group), potentially limiting individual contribution depth. | pp. 27–28 |
| **6** | **Describe any insights gained from mixing or integrating methods.** | Integration insights are presented throughout the Discussion. The qualitative findings contextualized and deepened the quantitative satisfaction scores — for example, explaining faculty dissatisfaction regarding research and infrastructure through FGD-identified themes of resource constraints and administrative burden. | pp. 23–27 |

| **COREQ — Consolidated Criteria for Reporting Qualitative Research (Tong et al., 2007, Int J Qual Health Care), 32-item checklist** | | | |
| --- | --- | --- | --- |
| **No.** | **COREQ Item** | **Where Addressed in Manuscript** | **Page** |
| **DOMAIN 1: Research Team and Reflexivity** | | | |
| **Personal Characteristics** | | | |
| **1** | **Interviewer/facilitator: Which author/s conducted the interview or focus group?** | Authors listed on title page; all are affiliated with Egyptian medical faculties involved in the national reform. The FGDs were facilitated by members of the research team. | p. 1 |
| **2** | **Credentials: What were the researcher's credentials, e.g. PhD, MD?** | Authors' credentials are stated in their affiliations (professors, program directors, quality assurance leaders). | p. 1 |
| **3** | **Occupation: What was their occupation at the time of the study?** | Faculty and medical education researchers at Egyptian medical colleges and Arabian Gulf University. | p. 1 |
| **4** | **Gender: Was the researcher male or female?** | Both male and female researchers (stated in Researcher Positionality section). | p. 10 |
| **5** | **Experience and training: What experience or training did the researcher have?** | Researchers have expertise in medical education, program evaluation, and qualitative research. | Implicit |
| **Relationship with Participants** | | | |
| **6** | **Relationship established: Was a relationship established prior to study commencement?** | Yes — several researchers were involved in implementing the national reform program, giving them established relationships with participating leaders. | p. 10 |
| **7** | **Participant knowledge of the interviewer: What did participants know about the researcher?** | Participants were aware that researchers were colleagues in the national medical education reform effort. This is addressed in the Researcher Positionality section. | p. 10 |
| **8** | **Interviewer characteristics: What characteristics were reported about the interviewer/facilitator?** | Insider positioning acknowledged; safeguards detailed in Researcher Positionality and Reflexivity subsection. | p. 10 |
| **DOMAIN 2: Study Design** | | | |
| **Theoretical Framework** | | | |
| **9** | **Methodological orientation and theory: What methodological orientation was stated to underpin the study?** | Thematic analysis following Braun & Clarke (2006) — inductive, data-driven approach. Evaluation framework: Kirkpatrick Model (behavior and results levels). | pp. 7, 11 |
| **Participant Selection** | | | |
| **10** | **Sampling: How were participants selected?** | Comprehensive purposive sampling: all key academic leaders from all 27 medical colleges were invited — deans, vice deans, program directors, and quality assurance unit heads. | pp. 6–7 |
| **11** | **Method of approach: How were participants approached?** | Invited through official institutional channels, as part of the national reform evaluation process. | p. 7 |
| **12** | **Sample size: How many participants were in the study?** | 100 participants across 9 FGDs (46 males, 54 females). Demographic breakdown reported in Section 3 (Results). | pp. 7, 17 |
| **13** | **Non-participation: How many people refused to participate or dropped out? Reasons?** | Not reported. All invited leaders participated; no refusals noted. | N/A |
| **Setting** | | | |
| **14** | **Setting of data collection: Where was the data collected?** | FGDs conducted across participating medical colleges; timing December 2023–December 2024. | p. 7 |
| **15** | **Presence of non-participants: Was anyone else present besides the researchers and participants?** | Not reported. | N/A |
| **16** | **Description of sample: What are the important characteristics of the sample?** | Demographic breakdown in Section 3: academic rank (75% professors, 25% associate professors), administrative roles (deans 25%, vice deans 27%, program directors 22%, QA directors 26%), gender (46% male, 54% female). | p. 17 |
| **Data Collection** | | | |
| **17** | **Interview guide: Were questions, prompts, guides provided by the authors?** | Yes — semi-structured FGD guide prepared by the research team; topics included implementation, challenges, and recommendations. | p. 9 |
| **18** | **Repeat interviews: Were repeat interviews carried out? If yes, how many?** | No. Single FGD per group of colleges. | N/A |
| **19** | **Audio/visual recording: Did the research use audio or visual recording to collect the data?** | Yes — sessions were audio-recorded after obtaining consent from all participants. | p. 9 |
| **20** | **Field notes: Were field notes made during and/or after the interview or focus group?** | Not explicitly stated. An audit trail was maintained during thematic analysis. | p. 11 |
| **21** | **Duration: How long were the interviews or focus group?** | 45–60 minutes per FGD. | p. 17 |
| **22** | **Data saturation: Was data saturation discussed?** | Yes — recurring themes were observed across later FGDs; no major new concepts emerged in subsequent discussions. | p. 7 |
| **23** | **Transcripts returned: Were transcripts returned to participants for comment and/or correction?** | Not reported. | N/A |
| **DOMAIN 3: Analysis and Findings** | | | |
| **Data Analysis** | | | |
| **24** | **Number of data coders: How many data coders coded the data?** | Multiple researchers independently coded the data; discrepancies resolved through discussion and consensus. | p. 11 |
| **25** | **Description of coding tree: Did authors provide a description of the coding tree?** | Themes and sub-themes presented in Table 4 (thematic analysis table). | pp. 52–55 |
| **26** | **Derivation of themes: Were themes identified in advance or derived from the data?** | Inductive approach — themes emerged from the data following Braun & Clarke's six phases. | p. 11 |
| **27** | **Software: What software, if applicable, was used to manage the data?** | No software — analysis conducted manually. | p. 11 |
| **28** | **Participant checking: Did participants provide feedback on the findings?** | Not reported. | N/A |
| **Reporting** | | | |
| **29** | **Quotations presented: Were participant quotations presented to illustrate the themes/findings?** | Yes — representative verbatim quotes presented for each theme/sub-theme in Section 3 (Qualitative Analysis). | pp. 18–22 |
| **30** | **Data and findings consistent: Was there consistency between the data presented and the findings?** | Yes — quotes are linked to specific themes in Table 4 and narrated in Section 3. | pp. 17–22 |
| **31** | **Clarity of major themes: Were major themes clearly presented in the findings?** | Yes — four major themes with sub-themes and codes presented in Table 4 and narrated in full in Section 3. | pp. 17–22 |
| **32** | **Clarity of minor themes: Is there a description of diverse cases or discussion of minor themes?** | Minority or dissenting views (e.g., initial student negativity, some faculty resistance to new methods) are acknowledged within the themes. Reflexivity statement documents commitment to including disconfirming perspectives. | pp. 10–11, 19–21 |

*References:*

- *O'Cathain A, Murphy E, Nicholl J. The quality of mixed methods studies in health services research. J Health Serv Res Policy. 2008;13(2):92–8.*
- *Tong A, Sainsbury P, Craig J. Consolidated criteria for reporting qualitative research (COREQ): a 32-item checklist for interviews and focus groups. Int J Qual Health Care. 2007;19(6):349–57.*
